# Diagnostic Power of Head-Up Tilt Test Enhanced by Autonomic ECG Parameters and Beat-to-Beat Hemodynamic Monitoring

**DOI:** 10.1155/crp/5239630

**Published:** 2025-05-15

**Authors:** Mathias Klemm, Antonia Kellnar, Dominik Naumann, Stefan Brunner, Christopher Stremmel

**Affiliations:** Medizinische Klinik und Poliklinik I, LMU Klinikum, Munich, Bavaria, Germany

**Keywords:** autonomic function, blood pressure, noninvasive monitoring, syncope, tilt test

## Abstract

**Objectives:** The head-up tilt test (HUTT) is a well-established diagnostic procedure used to differentiate between the types of syncope. Since its introduction in 1986, the protocol has undergone several refinements aimed at increasing diagnostic accuracy. Despite growing interest in advanced autonomic ECG parameters and beat-to-beat blood pressure monitoring, their integration into routine HUTT protocols remains limited.

**Methods:** In this study, we compared the conventional HUTT protocol using two-minute interval monitoring with an advanced protocol incorporating autonomic ECG parameters—periodic repolarization dynamics (PRD) and deceleration capacity (DC)—as well as continuous beat-to-beat hemodynamic monitoring.

**Results:** The extended protocol improves diagnostic resolution by detecting more pronounced hemodynamic fluctuations, enabling real-time trend analysis, and allowing earlier recognition of impending syncope. The tilt phase was characterized by a significant initial increase in PRD, and patients with syncope showed significantly higher PRD values during the tilt phase (8.14 vs. 3.91 deg^2^, *p*=0.043).

**Conclusions:** Continuous hemodynamic monitoring during HUTT improves the diagnostic quality by detecting changes at an early stage, thus allowing to anticipate syncope and to clearly identify its etiology. While beat-to-beat blood pressure monitoring is already recommended by current syncope guidelines, we propose the additional evaluation of autonomic ECG parameters as a valuable extension to standard protocols.

## 1. Introduction

Syncope is defined as a transient loss of consciousness due to transient global cerebral hypoperfusion. Although a sudden drop in systemic blood pressure (BP) is commonly the immediate trigger, identifying the underlying pathophysiological mechanism remains challenging. The head-up tilt test (HUTT) is routinely employed to detect various forms of autonomic dysregulation in patients with recurrent syncope or presyncope [1, 2]. In its original publication in 1986, Kenny and coworkers exposed the patient to a 40° tilt for 60 min to provoke vasovagal syncope [3, 4]. In the following years, this original protocol was modified to enhance sensitivity and reduce duration. According to the 2018 ESC guidelines and the 2017 ACC/AHA/HRS guidelines on syncope, HUTT is recommended with a Class IIa level B indication for evaluating suspected reflex syncope, orthostatic hypotension, and psychogenic pseudosyncope [1, 2].

Despite its widespread use, diagnostic limitations persist. We hypothesized that the integration of high-resolution ECG-derived autonomic markers—namely, periodic repolarization dynamics (PRD) and deceleration capacity (DC)—could optimize HUTT performance and facilitate pathophysiological understanding in patients with unexplained syncope. PRD quantifies low-frequency sympathetic modulation of ventricular repolarization, while DC reflects parasympathetic influence via analysis of RR-interval dynamics [5–8]. In addition, beat-to-beat BP monitoring, as suggested by the current ESC and ACC/AHA/HRS guidelines (Class IIb), can capture rapid hemodynamic changes often missed by interval measurements, potentially increasing the diagnostic yield [1, 2]. Therefore, we included continuous noninvasive BP monitoring and the abovementioned ECG parameters in our advanced testing protocol to evaluate their optimization potential during HUTT.

## 2. Methods

### 2.1. Patients

In this prospective pilot study, we aimed to optimize the standard-of-care HUTT protocol by incorporating continuous recording of autonomic ECG parameters and beat-to-beat BP monitoring. We enrolled 22 patients with suspected autonomic syncope between March 2019 and November 2023. In accordance with the Declaration of Helsinki and German data protection laws, all patients in this analysis provided informed consent, and the study was approved by the local ethics committee (approval code: 370-16, LMU Munich, Germany).

### 2.2. Study Definition and Endpoints

Orthostatic syncope is defined as a progressive fall of 20 mmHg or more in systolic BP, or 10 mmHg or more in diastolic BP after a few minutes of standing or a 60° tilt. Syncope occurring within 3 min is commonly defined as classic orthostatic hypotension, and syncope occurring after more than 3 min is defined as delayed orthostatic hypotension [1]. Reflex syncope is characterized by a rapid decrease in both BP and heart rate, usually triggered externally by pain, emotion, or orthostatic stress, and often begins with prodromal symptoms such as a feeling of warmth, abdominal discomfort, or nausea. According to the Vasovagal International Study classification, there are three main forms: Type 1 is a mixed form with a significant drop in BP followed by a decrease in heart rate; Type 2, the cardioinhibitory form, is characterized by a predominant drop in heart rate to less than 40 beats per minute (bpm) for at least 10 s, or asystole for more than 3 s; Type 3, the vasodepressor form, features a predominant drop in BP while the heart rate remains stable [9]. Classification into these types of syncope was performed by five independent cardiologists. In cases of disagreement, the final classification was determined by a majority vote.

### 2.3. Data Collection

Baseline data were collected as part of the routine diagnostic and treatment protocol, and the indication for HUTT was independent of this study. During HUTT, we performed automated interval BP monitoring every two minutes and continuous ECG recording (Philips IntelliVue X2) for a total of 60 min according to the standard of care protocol: After 15 min, patients were transferred to a 60° tilt for up to 45 min. If syncope did not occur spontaneously, 0.4 mg of sublingual glyceryl trinitrate was administered. The test was terminated at the onset of (pre)syncope or after 60 min. In parallel, but not as an indicator of test termination, noninvasive continuous BP monitoring (Finapres, Nova, the Netherlands) and high-resolution ECG signals (TMSi Porti, the Netherlands) were recorded for retrospective evaluation of the potential for test optimization.

The ECG signals were recorded at 2000 Hz using Frank leads. The DC and PRD were calculated from these signals for the baseline (horizontal position) and tilt phase of HUTT using our proprietary SMARTlab software (Version 1.5.4). In addition, to increase temporal resolution, we calculated short-term DC and PRD from one-minute ECG segments using a sliding window.

DC is a measure of heart rate variability. It is calculated from RR-intervals using phase-rectified signal averaging to isolate vagal modulations of the heart rate. PRD is a measure of sympathetic influence at the myocardial level. It analyzes the beat-to-beat variability of the T-wave repolarization vector and quantifies periodic sympathetic modulations at frequencies below 0.1 Hz.

### 2.4. Statistical Analysis

Statistical analysis was performed using R 4.3.1 and Prism 9 (GraphPad, USA) software. Continuous variables were reported as median and interquartile range (IQR). The Wilcoxon rank sum test was used to compare groups, with *p* values < 0.05 considered statistically significant.

## 3. Results

### 3.1. Baseline Characteristics

Twenty-two patients with a history of syncope and an indication for HUTT were enrolled in this study. Ten patients (45.5%) were female with a median age of 52 years and a BMI of 23.9 kg/m^2^. Left ventricular ejection fraction was normal in all patients, among them, one of whom had hypertrophic cardiomyopathy. Coronary artery disease was present in three of 22 patients (13.6%). The baseline BP was 123/75 mmHg with a heart rate of 78 bpm. Regarding preexisting cardiac arrhythmias, three patients had atrial fibrillation, and one patient had a history of cardiac arrest. The reported conduction disorders were left bundle branch block in one patient and 1st degree atrioventricular block in another (Table 1).

### 3.2. Beat-to-Beat Monitoring Improves HUTT Resolution

**Figure 1:**
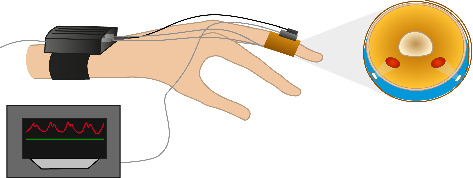
Continuous noninvasive blood pressure monitoring.

In addition to the conventional HUTT protocol, we performed continuous noninvasive hemodynamic monitoring using the Finapres NOVA device (Finapres Medical Systems, the Netherlands; Figure 1) to evaluate its potential advantages over standard interval BP measurements. Compared to standard interval BP recordings, beat-to-beat monitoring revealed a broader spectrum of systolic and diastolic values, allowing for earlier and more precise detection of hemodynamics. Systolic peaks were higher and nadirs lower, confirming the added sensitivity of continuous monitoring (Table 2).

### 3.3. Syncope Classification

The median duration of HUTT was 53 min. The primary endpoint, consisting of presyncope and syncope, was observed in two (9.1%) and ten (45.5%) patients, respectively, after a median study duration of 41 min. Five independent cardiologists classified the cause of syncope as reflex syncope Type 1 (mixed type) in three cases (13.6%), reflex syncope Type 2 (cardioinhibitory type) in five cases (22.7%), reflex syncope Type 3 (vasodepressor type) in two cases (9.1%), and as orthostatic syncope in two cases (9.1%) (Table 3).

### 3.4. Continuous BP Assessment Improves HUTT Quality

**Figure 2:**
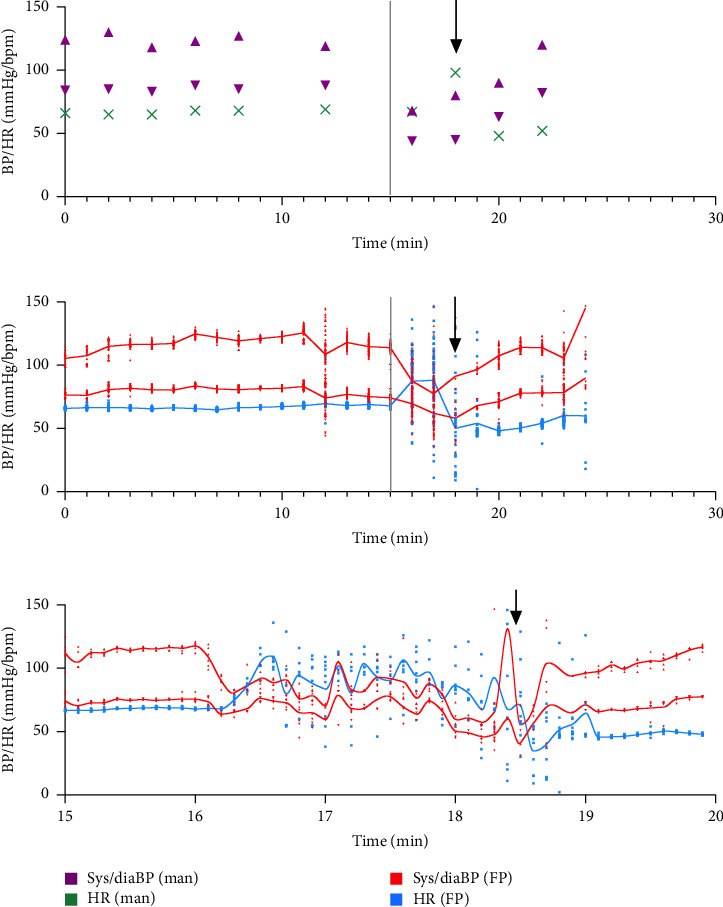
Graphical illustration of a representative examination. Depicted is a representative examination for reflex syncope type 2 with manual interval measurements (upper panel) and continuous Finapres NOVA (Finapres medical systems, the Netherlands) measurements (middle and lower panels). Gray vertical lines illustrate the tilting time point (after 15 min); black vertical arrows indicate the time point of syncope. BP, blood pressure; sys, systolic; dia, diastolic; HR, heart rate; man, manual; FP, finapres.

The recordings of the syncopal event itself also showed lower nadirs in continuous versus interval measurements (Tables 3 and 4). When comparing the cohorts based on the study endpoint, patients with syncope had significantly lower systolic BP and heart rate at the time point of syncope in both interval and continuous recordings (Table 4). Graphical data presentation was used to illustrate our findings (Figure 2).

### 3.5. Syncope and Autonomic ECG Parameters

To further strengthen our HUTT setup, we also evaluated Frank leads recordings to assess ECG parameters reflecting autonomic function, namely, PRD and DC. These parameters have been evaluated for cardiac risk prediction and are considered useful tools for studying events related to the autonomic nervous system [5–8]. Only 18 of 22 patients in our study population could be analyzed for autonomic ECG parameters due to insufficient quality and a corrupted data file. Two of the excluded patients had syncope.

The median baseline PRD value in our study cohort was 4.14 (2.07; 7.02) deg^2^. PRD was numerically higher during the tilt phase at 5.82 (3.66; 8.30) deg^2^ (*p*=0.182), with a significant increase in PRD during the first minutes of the tilt phase independent of syncope events (*p*=0.015 for Minute 1, *p*=0.002 for Minute 2, and *p*=0.004 for Minute 3). PRD during the entire tilt phase was significantly higher in patients with syncope (8.14 (6.36; 10.60) deg^2^) compared to patients without syncope (3.91 (3.64; 4.63) deg^2^, *p*=0.043) (Table 5).

The median baseline DC was 5.63 (3.56; 7.77) ms. There was a numerical trend toward lower DC values during the tilt phase, with a median of 4.58 (1.58; 7.56) ms (*p*=0.212) for the entire cohort (Table 5). Patients with (pre)syncope showed a nonsignificant increase in PRD (ΔPRD with syncope: +0.77 vs. ΔPRD without syncope: +0.08; *p*=0.360) as well as a decrease in DC in the tilt position (ΔDC with syncope: −1.73 vs. ΔDC without syncope: +0.30; *p*=0.068), independent of the type of syncope.

## 4. Discussion

In this study, we demonstrate the added potential of an advanced noninvasive monitoring system during HUTT. Continuous BP monitoring allows for earlier detection of impending syncope and increases the awareness of the attending physician immediately prior to the event. In addition, BP and heart rate values are more accurate due to very short recording intervals (i.e., more than one data point per second) and repetitive measurements. While the interval method carries the risk of potentially missing the most extreme measurements, this problem can be overcome by continuous recording. In addition, the classification of syncope types becomes much easier compared to the classical interval method. Especially in the very low BP range around the time of syncope, automated interval BP measurements may fail and important information is irretrievably lost. Even when compared to intrabrachial invasive BP measurements, Finapres measurements provided reliable results [10].

In the future, it may be possible to identify clear cutoffs for rapid BP changes sufficient to make a reliable diagnosis while avoiding the unpleasant event of complete loss of consciousness. Similarly, a study by Pitzalis et al. identified the first 15 min of tilt as having the highest predictive potential, which could be further enhanced by continuous recording [11]. The clear advantage of beat-to-beat BP monitoring is also reflected by ESC and ACC/AHA/HRS guideline recommendations, and we strongly suggest a further increase in the use of this technology worldwide.

It is important to note at this point that the concept of autonomic cardiac function analysis via ECG parameters has been present in the literature for several years. Even if the parameters PRD and DC analyzed here were not the focus at that time, the general concept has already proven to be very promising in HUTT and is further confirmed by our work [12–16]. For example, prior studies by Porta and colleagues highlighted the relevance of combined ventricular repolarization duration and heart period correlations for assessing autonomic function [13, 14].

PRD (sympathetic nervous system) and DC (parasympathetic nervous system) baseline values did not correlate with the occurrence of syncope. In general, our population had PRD and DC values considered physiological in previous studies [5, 6]. However, patients with syncope showed significantly higher PRD values in the tilt position, indicating the presence of autonomic dysregulation prior to syncope. All patients showed a significant increase in PRD during the first minutes of tilt, with no significant difference in PRD for the entire tilt phase. This suggests that patients who experience syncope during the tilt test exhibit more sustained sympathetic dysregulation than those without. Our study thus contributes novel insights by demonstrating the feasibility and clinical utility of integrating these parameters with noninvasive beat-to-beat BP monitoring.

Additionally, our methodology enables estimation of baroreflex sensitivity (BRS) through spontaneous fluctuations in RR-intervals and BP, as described in previous studies [17, 18]. While BRS was not a focus of this pilot study, we acknowledge its potential as a valuable metric in future research aimed at refining syncope diagnostics. Furthermore, characterizing HUTT responses could benefit from incorporating respiratory-related autonomic markers. Cardiorespiratory coupling, such as respiratory sinus arrhythmia or spectral components of RR-variability at the respiratory frequency, offers an additional window into vagal function. Although our setup did not include respiratory signal acquisition, future studies should consider integrating such measures to provide a more comprehensive assessment of autonomic cardiovascular regulation [19].

Our study is clearly limited by its sample size. However, it was designed as a pilot study to evaluate the feasibility of evaluating autonomic ECG parameters during HUTT. The current methods for calculating PRD and DC have been validated for measurements of at least 20 min. Therefore, some of the standing ECG recordings of patients with syncope are shorter than the usual recordings used. The error caused by artifacts, i.e., caused by movement, is greater in these measurements. Overall, the data quality in our study cohorts was good and promising for further evaluation in larger studies including these short-frame recordings.

## 5. Conclusions

The integration of autonomic ECG markers with beat-to-beat BP monitoring during HUTT is feasible, cost-effective, and diagnostically beneficial. Our findings support the inclusion of PRD and DC into clinical HUTT protocols and highlight future opportunities for autonomic profiling through BRS and respiratory-linked metrics. Larger, multicenter studies are warranted to validate these results and define clear diagnostic thresholds.

## Figures and Tables

**Table 1:** Baseline characteristics.

Age (years)	52 (34; 73)
Female sex (*n*, %)	10 (45.5)
BMI (kg/m^2^)	23.9 (23.0; 27.3)
Ejection fraction (%)	60 (60; 60)
Cardiomyopathy (*n*, %)	1 (4.6%): HCM
CAD (*n*, %)	3 (13.6%)
Neurological disorder (*n*, %)	0 (0%)
Systolic BP (mmHg)	125 (110; 134)
Diastolic BP (mmHg)	75 (70; 80)
Heart rate (bpm)	78 (65; 82)
Atrial fibrillation (*n*, %)	3 (13.6)
History of cardiac arrest (*n*, %)	1 (4.6)
AV block 1 (*n*, %)	1 (4.6)
AV block ≥ 2 (*n*, %)	0 (0)
BBB (*n*, %)	1 (4.6): LBBB

*Note:* Values are presented as median (IQR) if not indicated otherwise. *N* = 22.

Abbreviations: AV = atrioventricular, BMI = body mass index, CAD = coronary artery disease, (L)BBB = (left) bundle branch block.

**Table 2:** Interval vs. continuous monitoring during HUTT.

	Interval	Continuous	*p* value
Max. systolic BP	135 (121; 150)	168 (139; 211)	**< 0.001**
Max. diastolic BP	80 (80; 89)	111 (98; 125)	**< 0.001**
Max. heart rate	106 (95; 120)	119 (101; 134)	**0.003**
Min. systolic BP	98 (81; 108)	82 (68; 98)	**0.018**
Min. diastolic BP	60 (50; 70)	60 (46; 66)	0.246
Min. heart rate	60 (53; 72)	47 (37; 60)	**< 0.001**

*Note:* Units are mmHg for blood pressure and bpm for heart rate. Values are presented as median (IQR). *N* = 22. Max = maximum, min = minimum.

Abbreviation: BP = blood pressure.

**Table 3:** Syncope classification.

Duration (min)	53 (45; 60)
Time until syncope (min)	41 (29; 51)
Reflex syncope, Type 1 [mixed] (*n*, %)	3 (13.6)
Reflex syncope, Type 2 [cardioinhibitory] (*n*, %)	5 (22.7)^∗^
Reflex syncope, Type 3 [vasodepressor] (*n*, %)	2 (9.1)
Orthostatic syncope (*n*, %)	2 (9.1)

*Note:* Values are presented as median (IQR) if not indicated otherwise. *N* = 22.

^∗^One pat. with AVB 3, one pat. with asystole.

**Table 4:** Hemodynamic characteristics with and without syncope.

		Syncope (*N* = 12)	No syncope (*N* = 10)	*p* value
Interval	Max. systolic BP	135 (129; 145)	143 (120; 150)	0.961
	Max. diastolic BP	83 (80; 90)	80 (80; 84)	0.585
	Max. heart rate	109 (94; 116)	103 (95; 120)	0.935
	Min. systolic BP	83 (68; 93)	105 (100; 110)	**0.002**
	Min. diastolic BP	55 (40; 61)	70 (61; 74)	**0.020**
	Min. heart rate	55 (52; 60)	73 (67; 80)	**0.002**
	Syncope systolic BP^∗^	75 (58; 88)	—	—
	Syncope diastolic BP^∗^	51 (40; 62)	—	—
	Syncope heart rate^∗^	63 (40; 67)	—	—

Continuous	Max. systolic BP	174 (152; 215)	155 (123; 196)	0.486
	Max. diastolic BP	111 (104; 121)	107 (97; 142)	0.734
	Max. heart rate	119 (109; 134)	117 (95; 133)	0.426
	Min. systolic BP	68 (58; 81)	96 (86; 106)	**0.003**
	Min. diastolic BP	46 (43; 63)	65 (59; 69)	**0.016**
	Min. heart rate	41 (21; 47)	59 (50; 68)	**0.006**
	Syncope systolic BP	68 (58; 81)	—	—
	Syncope diastolic BP	46 (43; 63)	—	—
	Syncope heart rate	41 (21; 47)	—	—

*Note:* Units are mmHg for blood pressure and bpm for heart rate. Values are presented as median (IQR). Bold *p* values indicate statistical significance. Max = maximum, min = minimum.

Abbreviation: BP = blood pressure.

^∗^For interval measurements, the data at the time of syncope refer to the last value recorded before the event.

**Table 5:** ECG parameters of autonomic function.

	Total (*N* = 18)	Syncope (*N* = 10)	No syncope (*N* = 8)	*p* value
PRD baseline	4.14 (2.07; 7.02)	5.52 (2.78; 8.54)	3.23 (1.79; 6.46)	0.360
PRD tilt	5.82 (3.66; 8.30)	8.14 (6.36; 10.60)	3.91 (3.64; 4.63)	**0.043**
DC baseline	5.63 (3.56; 7.77)	7.41 (4.17; 8.13)	4.95 (3.18; 6.09)	0.274
DC tilt	4.58 (1.58; 7.56)	4.83 (1.44; 7.56)	4.58 (2.70; 7.09)	0.965
PRD Tilt Minute 1	11.50 (8.88; 18.40)	14.42 (9.38; 31.59)	10.78 (8.34; 11.98)	0.274
PRD Tilt Minute 2	11.31 (9.86; 21.50)	20.31 (10.85; 26.93)	10.41 (8.38; 13.28)	0.173
PRD Tilt Minute 3	10.13 (8.49; 29.25)	10.13 (8.97; 30.01)	9.67 (7.05; 14.83)	0.236
DC Tilt Minute 1	4.48 (2.60; 9.93)	6.89 (2.60; 12.11)	4.48 (3.01; 6.62)	0.573
DC Tilt Minute 2	5.57 (3.65; 8.48)	4.9 (3.65; 7.42)	7.65 (3.38; 8.59)	0.743
DC Tilt Minute 3	6.82 (2.54; 8.33)	6.96 (5.07; 7.51)	6.02 (1.02; 8.44)	0.879

*Note:* Units are deg^2^ for PRD and ms for DC. Values are presented as median (IQR). Bold *p* values indicate statistical significance.

Abbreviations: DC = deceleration capacity, PRD = periodic repolarization dynamics.

## Data Availability

The data that support the findings of this study are available from the corresponding author upon reasonable request.
